# Pharmacological Inhibition and Activation of the Ca^2+^ Activated Cl^−^ Channel TMEM16A

**DOI:** 10.3390/ijms21072557

**Published:** 2020-04-07

**Authors:** Raquel Centeio, Inês Cabrita, Roberta Benedetto, Khaoula Talbi, Jiraporn Ousingsawat, Rainer Schreiber, John K. Sullivan, Karl Kunzelmann

**Affiliations:** 1 Physiological Institute, University of Regensburg, D-93053 Regensburg, Germany; raquel.martins-centeio@vkl.uni-regensburg.de (R.C.); ines.cabrita@vkl.uni-regensburg.de (I.C.); Roberta.Benedetto@ur.de (R.B.); khaoula.talbi@vkl.uni-regensburg.de (K.T.); jiraporn.ousingsawat@vkl.uni-regensburg.de (J.O.); Rainer.Schreiber@vkl.uni-regensburg.de (R.S.); 2 Æon Respire, Inc., Newbury Park, CA 91320, USA; jksullivan@aeonrespire.com

**Keywords:** TMEM16A, TMEM16F, benzbromarone, niclosamide, Ani9, TMEM16A potentiators, Eact, PIP_2_

## Abstract

TMEM16A is a Ca^2+^ activated Cl^−^ channel with important functions in airways, intestine, and other epithelial organs. Activation of TMEM16A is proposed as a therapy in cystic fibrosis (CF) to reinstall airway Cl^−^ secretion and to enhance airway surface liquid (ASL). This CFTR-agnostic approach is thought to improve mucociliary clearance and lung function in CF. This could indeed improve ASL, however, mucus release and airway contraction may also be induced by activators of TMEM16A, particularly in inflamed airways of patients with asthma, COPD, or CF. Currently, both activators and inhibitors of TMEM16A are developed and examined in different types of tissues. Here we compare activation and inhibition of endogenous and overexpressed TMEM16A and analyze potential off-target effects. The three well-known blockers benzbromarone, niclosamide, and Ani9 inhibited both TMEM16A and ATP-induced Ca^2+^ increase by variable degrees, depending on the cell type. Niclosamide, while blocking Ca^2+^ activated TMEM16A, also induced a subtle but significant Ca^2+^ store release and inhibited store-operated Ca^2+^ influx. Niclosamide, benzbromarone and Ani9 also affected TMEM16F whole cell currents, indicating limited specificity for these inhibitors. The compounds Eact, cinnamaldehyde, and melittin, as well as the phosphatidylinositol diC8-PIP_2_ are the reported activators of TMEM16A. However, the compounds were unable to activate endogenous TMEM16A in HT_29_ colonic epithelial cells. In contrast, TMEM16A overexpressed in HEK293 cells was potently stimulated by these activators. We speculate that overexpressed TMEM16A might have a better accessibility to intracellular Ca^2+^, which causes spontaneous activity even at basal intracellular Ca^2+^ concentrations. Small molecules may therefore potentiate pre-stimulated TMEM16A currents, but may otherwise fail to activate silent endogenous TMEM16A.

## 1. Introduction

The Ca^2+^-activated Cl^−^ channel (CaCC) TMEM16A is expressed in epithelial and non-epithelial cells where it contributes to a broad range of cellular functions [1]. TMEM16A produces fast gating single channel currents of very small amplitude. TMEM16A whole cell currents are voltage dependent, slowly activating, and outwardly rectifying. The channel conducts anions according to the *Eisenman* type I selectivity sequence. In airways TMEM16A is sparsely expressed in surface epithelial cells with somewhat larger expression in submucosal serous and mucous producing goblet cells (summarized in [2]). TMEM16A is also expressed in airway smooth muscle (ASM) [3,4,5,6]. In both asthma and cystic fibrosis (CF), and upon exposure of airway epithelial cells to bacterial components, TMEM16A is strongly upregulated, particularly in cells of submucosal glands [7,8,9]. In CF, impaired function of the cystic fibrosis transmembrane conductance regulator (CFTR) leads to a defect in epithelial Cl^−^ secretion, causing reduced airway surface liquid (ASL) with the consequence of a dehydrated sticky mucus and possibly low ASL pH (discussed in [2]. Pharmacological activation of TMEM16A is thought to compensate for the absent CFTR-dependent Cl^−^ secretion in CF, and therefore may represent a CFTR mutation-agnostic therapy [10]. Other studies found a role of TMEM16A for ASM contraction [6,9,11]. Such a role of TMEM16A is particularly evident under inflammatory conditions like asthma, when TMEM16A is strongly upregulated in the ASM. Because asthma appears to be a common problem also in patients with CF [12], therapeutic activation of TMEM16A could trigger bronchoconstriction in CF patients. Activation of TMEM16A could also trigger mucus release and enhance airway mucus plugging [3,5,13,14].

In order to elucidate the contribution of TMEM16A to different airway functions and to define their role in other tissues, pharmacological compounds, i.e., small molecule inhibitors and activators of TMEM16A are frequently used. A relatively large number of small molecules have been found to inhibit TMEM16A. These molecules are surprisingly diverse in structure and do not specifically inhibit only TMEM16A [2]. Similarly, quite different molecules were reported to activate TMEM16A [2], with controversial reports on their efficacy and mechanism of action. Some molecules, such as the putative TMEM16A-activator Eact, were suspected to increase intracellular Ca^2+^ rather than directly activating TMEM16A [15,16].

A recent report describes the effects of the novel TMEM16A potentiator ETX001 on fluid secretion in cultured airway epithelia cells, and on the mucociliary clearance in sheep trachea [17]. Currently it remains obscure how ETX001 potentiates TMEM16A activity. It will be interesting to learn more about the mechanism of action for ETX001, and to learn whether the compound shares some functional properties with other molecules, such as the TMEM16A-regulator phosphatidylinositol 4,5-bisphosphate (PIP_2_) [18,19]. Distinct binding sites for Ca^2+^ and PIP_2_ have been identified, with Ca^2+^ binding to transmembrane domains 6–8 leading to opening of the channel and PIP_2_ binding to domains 3–5 stabilizing the channel in the open configuration [19]. These results provide an insight into the process of Ca^2+^ desensitization, i.e., inactivation (run down) of the channel and give hints as to further developments of activators/potentiators of TMEM16A. Along this line, our own previous results described the activation and potentiation, respectively, of TMEM16A by the inositolphosphate INO-4995 [20].

The purpose of the present study was to compare effects of activators and inhibitors of TMEM16A on endogenous human (h)TMEM16A and hTMEM16A overexpressed in HEK293 cells. The study was triggered by our earlier findings showing a strong activation of overexpressed TMEM16A by the inositolphosphate INO-4995, but a more indirect effect of INO-4995 on endogenous TMEM16A expressed in colonic and airway epithelial cells [20]. Moreover, a basal activity at resting Ca^2+^ concentrations was found for overexpressed but not for endogenous TMEM16A [21]. In other studies, TMEM16A was found to control the magnitude of local, i.e., submembranous Ca^2+^ signals by interacting with the IP3 receptor [22,23]. Thus, TMEM16A modulates its own local environment in a way so that it is activated through locally concentrated Ca^2+^ release [24]. In many previous studies as well as ongoing work, inhibitors and activators of TMEM16A are used, although they may also affect other TMEM16 paralogs or may exert additional effects on Ca^2+^ regulating proteins [16,25]. Here we confirm the role of TMEM16A for intracellular Ca^2+^ signaling and show for a number of compounds that they also affect TMEM16F, a paralog of TMEM16A.

## 2. Results

### 2.1. Ca^2+^-Dependent Cl^−^ Transport in Colonic Epithelial Cells is not Activated by Eact

**Figure 1:**
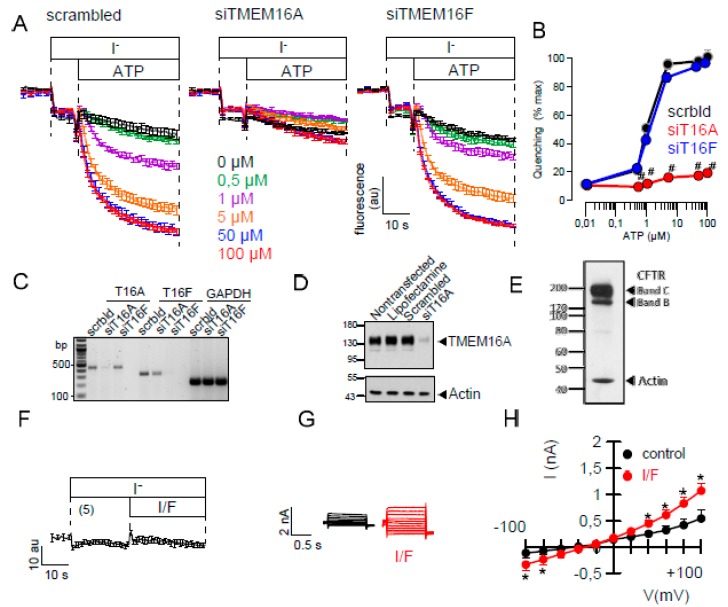
Endogenous Ca^2+^-dependent TMEM16A and cAMP-activated CFTR Cl^−^ transport in HT_29_ colonic epithelial cells. (**A**,**B**) YFP fluorescence quenching by iodide is enhanced by ATP in a concentration-dependent manner (*n* = 5 for all). siRNA knockdown of TMEM16A but not TMEM16F inhibits quenching. (**C**) Semiquantitative RT-PCR indicates knockdown of TMEM16A (T16A; 92%, *n* = 3) and TMEM16F (T16F; 93%, *n* = 3). (**D**,**E**) Western blotting indicating pronounced expression of endogenous TMEM16A and CFTR in HT_29_ cells. siRNA knocked down expression of TMEM16A. (**F**–**H**) Activation of chloride conductance by IBMX and forskolin (I/F; 100 µM/2 µM) was not detected in iodide quenching (*n* = 5), but was significant in whole cell patch clamp recordings (overlay currents and I/V curves; *n* = 5). Mean ± SEM. * significant activation (*p* < 0.05; paired *t*-test). ^#^ significant inhibition (*p* < 0.05; ANOVA).

**Figure 2:**
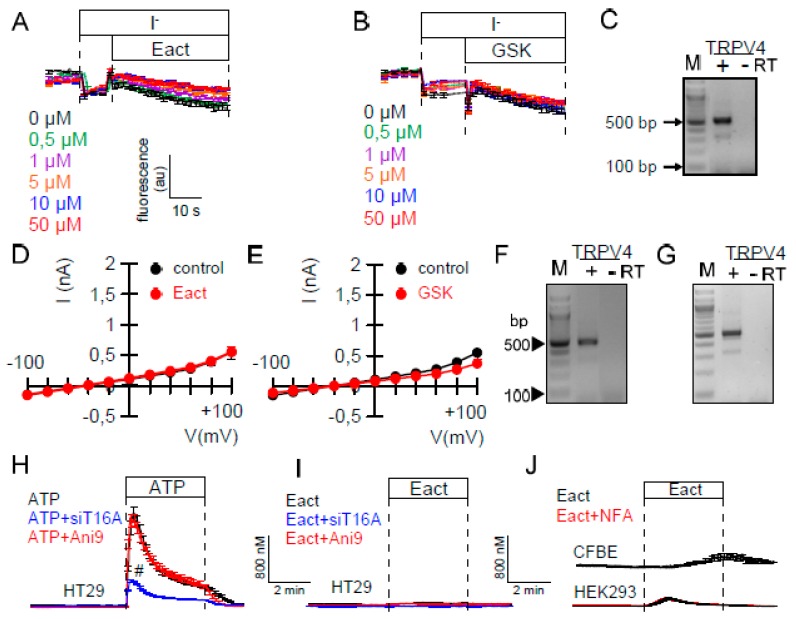
Eact does not activate endogenous Ca^2+^-dependent Cl^−^ secretion, and has little effect on [Ca^2+^]_i_. (**A**,**B**) No activation of iodide quenching by Eact or GSK1016790 (*n* = 5 for both). (**C**) RT-PCR indicating the expression of TRPV4 in HT_29_ cells. (**D**,**E**) No activation of whole cell currents in HT_29_ cells by Eact or GSK1016790 (10 µM; *n* = 5 for both). (**F**,**G**) RT-PCR of TRPV4 expressed in CFBE (**F**) and HEK293 (**G**) cells. (**H**) ATP (100 µM) induced [Ca^2+^]_i_ rise is inhibited by knockdown of TMEM16A but not by Ani9 (10 µM; *n* = 37–105). (**I**,**J**) Minor increase in [Ca^2+^]_i_ by Eact in HT_29_, CFBE, or HEK293 cells (*n* = 31–111). Mean ± SEM. ^#^ significant inhibition (*p* < 0.05; unpaired *t*-test).

Using different mouse knockout models, we previously demonstrated that Ca^2+^ dependent Cl^−^ secretion in mouse intestine requires TMEM16A, but not TMEM16F [26]. TMEM16A is a bona fide Ca^2+^ activated Cl^−^ channel, while TMEM16F is a Ca^2+^ activated phospholipid scramblase that also conducts ions [1,27,28,29]. Here we examined HT_29_ colonic epithelial cells stably expressing yellow fluorescent protein (kindly provided by Prof. Dr. Luis Galietta, Telethon Institute of Genetics and Medicine, Pozzuoli, Italy). We found that in HT_29_ cells, Cl^−^ secretion by ATP is entirely due to the activation of TMEM16A. It is independent of TMEM16F, as shown by siRNA-knockdown and measurement of I^-^ - induced YFP-quenching (Figure 1A–C). HT_29_ cells demonstrate strong expression of TMEM16A, which can be well downregulated by siRNA (Figure 1D). Also CFTR is well expressed in HT_29_ cells, but cAMP (I/F)-activated YFP quenching was slow and the activated whole cells currents were small (Figure 1E–H). The compounds Eact [15] and GSK1016790, both activators of Ca^2+^ permeable TRPV channels, did not activate TMEM16A in YFP-quenching experiments with HT_29_ cells, although TRPV4 was found to be well expressed in these cells (Figure 2A–C). Similarly, Eact or GSK1016790 did not increase whole cell currents in patch clamp experiments (Figure 2D,E). This may correspond to the results of a previous report, demonstrating Eact as an activator of Ca^2+^ permeable TRPV4 channels, rather than a direct activator of TMEM16A [16]. Although TRPV4 is expressed in HT_29_ cells, the rise in intracellular Ca^2+^ induced by Eact was insignificant, particularly when compared with the effect of ATP on intracellular Ca^2+^ ([Ca^2+^]_i_) (Figure 2H,I). To further validate the role of TRPV4/Eact for Ca^2+^ signaling, we also analyzed expression of TRPV4 and the increase in intracellular Ca^2+^ by Eact in CFBE airway epithelial cells and HEK293 cells (Figure 2F,G). In these two cell lines, Eact only marginally increased intracellular Ca^2+^, although TRPV4 was found to be well expressed (Figure 2F,G,I,J).

### 2.2. Effects of Inhibitors of TMEM16A on Whole Cell Currents and Intracellular Ca^2+^

**Figure 3:**
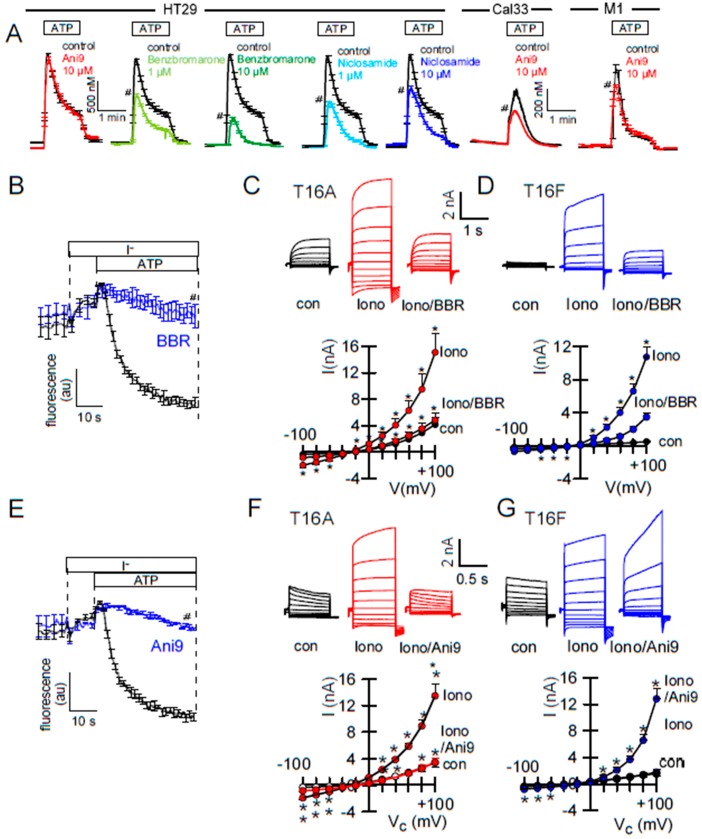
Effect of inhibitors on Ca^2+^-activated Cl^−^ transport and ATP-induced rise in [Ca^2+^]_i_**. A**) Increase in intracellular Ca^2+^ by stimulation of HT_29_ cells with ATP (100 µM). In contrast to benzbromarone and niclosamide, Ani9 did not attenuate the effect of ATP on [Ca^2+^]_i_ (*n* = 7–23). In Call33 head and neck cancer cells and M1 mouse collecting duct cells Ani9 (10 µM) inhibited ATP-induced Ca^2+^ increase (*n* = 134–162) significantly. **B**) Inhibition of ATP (5 µM) activated YFP-quenching in HT_29_ cells by BBR (10 µM). **C**) Inhibition of ionomycin (Iono, 1 µM) activated whole cell currents by BBR in HEK293 cells overexpressing TMEM16A (original recording and I/V curves) (*n* = 5–6). **D**) Inhibition of ionomycin (Iono; 1 µM) activated whole cell currents by BBR in HEK293 cells overexpressing TMEM16F (*n* = 6, original recording and I/V curves). **E**) Inhibition of ATP (5 µM) induced YFP-quenching in HT_29_ cells by Ani9. **F**,**G**) Inhibition of ionomycin (Iono; 1 µM) activated TMEM16A currents by Ani9 (10 µM), and change in time-dependent activation of TMEM16F currents (*n* = 5–8). * significant inhibition (*p* < 0.05; paired *t*-test). ^#^ significant inhibition (*p* < 0.05; unpaired *t*-test).

**Figure 4:**
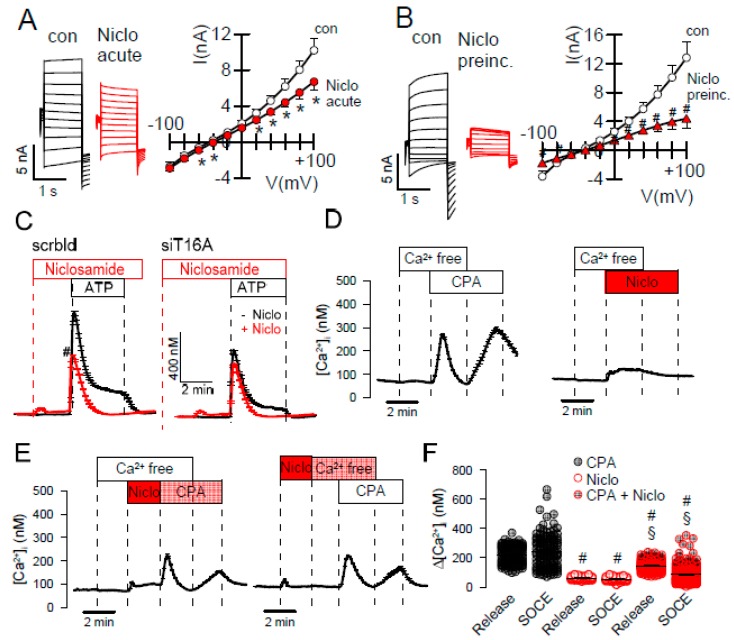
Niclosamide inhibits TMEM16A currents activated by intracellular Ca^2+^. **(A**) TMEM16A overexpressed in HEK293 cells was activated by 1 µM Ca^2+^ in the patch pipette filling solution. Acute application of niclosamide (Niclo; 5 µM) significantly inhibited Ca^2+^-activated TMEM16A whole cell currents (*n* = 7). (**B**) 15 min preincubation with Niclo inhibited TMEM16A more potently (*n* = 9–10). (**C**) Increase in intracellular Ca^2+^ with ATP (100 µM) in HT_29_ cells. Niclosamide (5 µM) induces a slight and transient increase in [Ca^2+^]_i_ and inhibits ATP-induced rise in [Ca^2+^]_i_. siRNA knockdown of TMEM16A inhibits increase in [Ca^2+^]_i_ by ATP. Niclosamide shows no additional effects on [Ca^2+^]_i_ (*n* = 60–193). (**D**) ER Ca^2+^ store release and Ca^2+^ influx (SOCE) induced by CPA and niclosamide (*n*= 40–50). (**E**,**F**) Effects of CPA and niclosamide on Ca^2+^ store release and SOCE under various conditions (*n* = 40–213) * significant inhibition (*p* < 0.05; paired *t*-test). ^#^ significant inhibition (*p* < 0.05; unpaired *t*-test).

In previous and the present study, inhibitors of TMEM16A attenuated receptor-mediated Ca^2+^ increase, supporting the role of TMEM16A Cl^−^ currents for Ca^2+^ store release (Figure 3A). On the other hand, physical interaction of TMEM16A with IP_3_-receptors was shown to augment local submembraneous Ca^2+^ increase [23,24]. This is also shown in the present study by siRNA-knockdown of TMEM16A: ATP-induced [Ca^2+^]_i_ increase was attenuated after siRNA knockdown of TMEM16A. Notably, after knockdown of TMEM16A, niclosamide had no further inhibitory effect on ATP-induced Ca2+-increase (Figure 4C). In contrast, inhibition of TMEM16A by the TMM16A-blocker Ani9 [30] did not attenuate Ca^2+^ signals, while it potently inhibited TMEM16A currents (Figure 3A,E,F). This suggests that tethering of IP_3_-receptors, but not TMEM16A currents, are relevant for modulation of intracellular Ca^2+^ levels. Many TMEM16A-inhibitors such as CaCCinhAO1, niflumic acid, niclosamide, and benzbromarone inhibited ATP-induced Ca^2+^ increase in the present and in previous studies (Figure 3A) [23,25]. Inhibition of intracellular Ca^2+^ signaling cold either mean that the inhibitors exert this effect through inhibition of TMEM16A, or they block Ca^2+^ regulating proteins independent of TMEM16A. Although Ani9 did not affect [Ca^2+^]_i_ in HT_29_ cells it showed some inhibition on intracellular Ca^2+^ in other cell types such as Cal33 and M1 mouse cells (Figure 3A). Depending on the expression of various Ca^2+^ regulating proteins in the different cell lines, side effects caused by non-specificity of small molecules might be variable in the different cell types. Both BBR and Ani9 potently inhibited TMEM16A activated in YFP-quenching and patch clamp experiments (Figure 3B,C,E,V). BBR also inhibited TMEM16F currents in HEK293 cells, while Ani9 changed the time dependence and inward currents of TMEM16F (Figure 3D,G). Thus, Ani9 appears to be more specific than other TMEM16A-inhibitors, but still affects ion currents and possibly gating properties of TMEM16F and maybe other TMEM16 paralogs.

Niclosamide was reported as potent inhibitor of TMEM16A that also inhibits TMEM16F [9,25]. Because niclosamide inhibits ATP-induced rise in [Ca^2+^]_i_ [25], we examined if the compound truly blocks TMEM16A channels directly and not or not only indirectly by affecting intracellular Ca^2+^ levels. To that end TMEM16A was activated by 1 µM Ca^2+^ in the patch pipette filling solution, to maintain a stably enhanced intracellular Ca^2+^ concentration. Under these conditions niclosamide either applied acutely or after preincubation clearly inhibited TMEM16A currents (Figure 4A,B). Inhibition of TMEM16A was more pronounced after short preincubation of the drug, suggesting that the rather lipophilic drug may accumulate in the plasma membrane which could affect TMEM16A-lipid interaction (Figure 4A,B). Inhibition of ATP-induced [Ca^2+^]_i_ increase by niclosamide was absent after siRNA-knockout of TMEM16A (Figure 4C). The SERCA-pump inhibitor cyclopiazonic acid (CPA) and, to a much lesser degree, niclosamide-induced ER Ca^2+^ store release (Figure 3D). Store operated Ca^2+^ entry (SOCE) after re-adding extracellular Ca^2+^ was not observed after niclosamide. The data suggest that niclosamide lowers Ca^2+^ store content and attenuates SOCE (Figure 3D,E). Thus, niclosamide does not only block TMEM16A and TMEM16F, but also inhibits the SERCA pump and Ca^2+^ influx channels. Many lipophilic small molecules and natural compounds are known to have these side effects. It remains currently unclear whether this is caused by a direct inhibition of Ca^2+^-transporting proteins or by a more non-specific membrane lipid interaction. These additional effects of niclosamide may contribute to the large number of its beneficial effects currently evaluated [31].

### 2.3. Activators of TMEM16A Act Differently on Endogenous and Overexpressed TMEM16A

**Figure 5:**
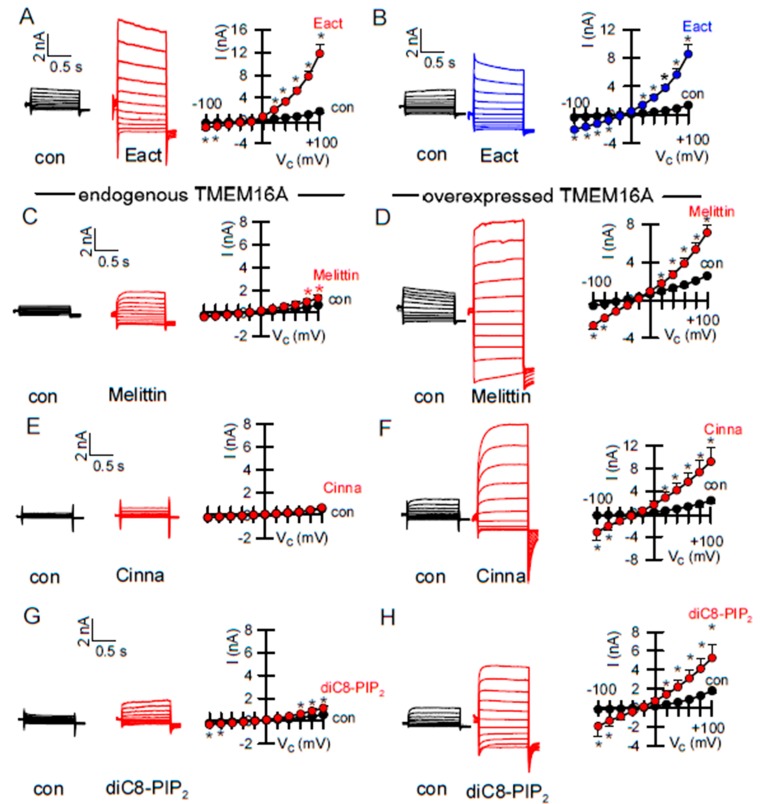
Endogenous and overexpressed TMEM16A behave differently. (**A**) Activation of overexpressed TMEM16A whole cell currents in HEK293 cells by Eact (10 µM, *n* = 9). (**B**) Activation of TMEM16F whole cell currents in TMEM16F-overexpressing HEK293 cells by Eact (10 µM, *n* = 6). (**C**,**D**) Little activation of endogenous TMEM16A currents by melittin (200 nM; *n* = 10) in HT29 cells, but strong activation in HEK293 cells overexpressing TMEM16A (*n* = 7). (**E**,**F**) Little activation of endogenous TMEM16A currents by cinnamaldehyde (Cinna; 1 µM; *n* = 9), but strong activation of overexpressed TMEM16A (*n* = 5). (**G**,**H**) Little activation of endogenous TMEM16A currents by diC8-PIP_2_ (50 µM; *n* = 6), but strong activation of overexpressed TMEM16A (*n* = 6). * significant activation (*p* < 0.05; paired *t*-test). ^#^ significant activation (*p* < 0.05; unpaired *t*-test).

Endogenous TMEM16A expressed in HEK293 cells is not activated by Eact (Figure 1). As outlined above, no immediate activation of endogenous TMEM16A was observed for INO-4995, while overexpressed TMEM16A was potently activated by INO-4995 [20]. Interestingly, we found a similar result for Eact, as it potently activated both TMEM16A and TMEM16F overexpressed in HEK293 cells (Figure 5A,B). Alerted by these results, we examined three diverse putative activators of TMEM16A, melittin [21], cinnamaldehyde [32], and diC8-PIP_2_ [19,33] (Table 1). All three compounds activated overexpressed TMEM16A (Figure 5D,F,H), but had little effect on endogenous TMEM16A (Figure 5C,E,G). In all patch clamp experiments pipette Ca^2+^ was clamped to 0.1 µM. The ability of different drugs to activate overexpressed TMEM16A may be due to a higher sensitivity of overexpressed channels for intracellular Ca^2+^. This is supported by the basal activity of overexpressed TMEM16A at a resting [Ca^2+^]i of 100 nM [21]. Overexpressed TMEM16A was also spontaneously active in YFP-quenching experiments. In contrast, currents were completely absent when [Ca^2+^]i was clamped to zero [21].

**Figure 6:**
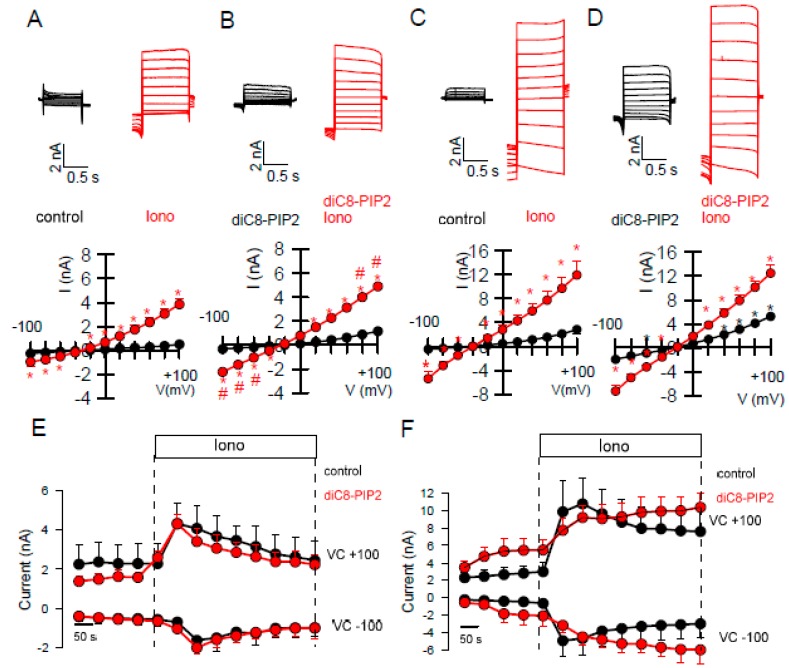
diC8-PIP2 augments TMEM16A currents activated by ionomycin. (**A**–**D**) Whole cell currents and I/V curves showing effect of diC8-PIP_2_ (50 µM in the patch pipette filling solution) on basal and ionomycin (Iono, 0.1 µM) activated TMEM16A currents in HT_29_ cells (**A**,**B**) and HEK293 cells (**C**,**D**). Activation of TMEM16A by diC8-PIP2 is clearly observed in TMEM16A-overexpressing HEK293 cells but not in HT_29_ cells (*n* = 6–7 for all). (**E**,**F**) Time courses for Iono-activated TMEM16A currents in HT_29_ and HEK293 cells (*n* = 6–8). * significant activation (*p* < 0.05; paired *t*-test). ^#^ significant difference to the absence of diC8-PIP_2_ (*p* < 0.05; unpaired *t*-test).

**Figure 7:**
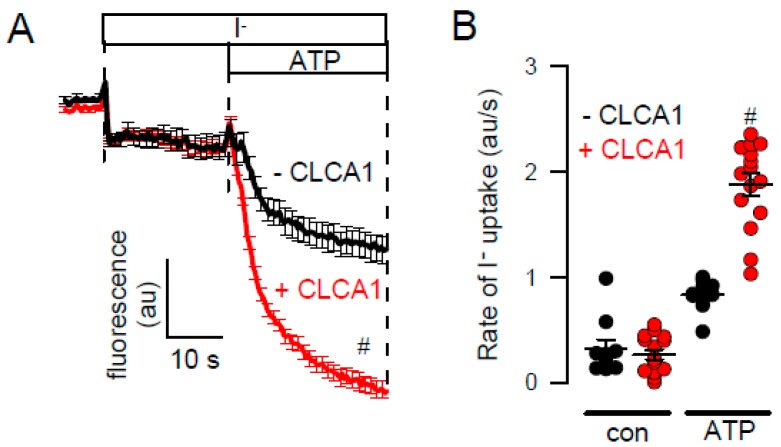
Increase in halide conductance by CLCA1. **A**,**B**) ATP (5 µM) induced iodide quenching of YFP in HT_29_ cells. Exposure of the cells for 24h to CLCA1-enriched media augmented ATP induced halide quenching. Mean ± SEM (*n* = 10–15). ^#^ significant difference when compared to the absence of CLCA1 (*p* < 0.05; unpaired *t*-test).

It has been reported that TMEM16A requires PIP_2_ to be opened by [Ca^2+^]_i_, and that PIP_2_ stabilizes the open conformation under high [Ca^2+^]_i_ [19]. We observed that endogenous TMEM16A currents activated by ionomycin were somewhat larger and more linear in the presence of diC8-PIP_2_ (Figure 6A,B). This may suggest that in the presence of PIP_2_, Ca^2+^ binding is facilitated. However, diC8-PIP_2_ did not delay the rundown of endogenous TMEM16A after ionomycin-stimulation (Figure 6E). In contrast to endogenous channels, overexpressed TMEM16A was clearly activated by diC8-PIP_2_ alone (Figure 6C,D). Rundown of the channel after stimulation with ionomycin was delayed in the presence of diC8-PIP_2_ (Figure 6C,D,F). Taken together, PIP_2_-dependent regulation is different for endogenous and overexpressed TMEM16A [18,19,20,34]. In contrast to the poorly understood mechanisms of the currently available activators of TMEM16A, such as Eact, cinnamaldehyde, ETX001, and others, the mechanism by which the TMEM16A-activator CLCA-1 increases membrane expression of TMEM16A has been described earlier [35]. CLCA1 is a secreted potentiator of the calcium-activated chloride channel. Its Willebrand factor type A domain elevates TMEM16A expression and activity within minutes, suggesting a dynamic association of TMEM16A with many partners that control its expression at various levels [36,37]. Thus, CLCA1 appears to work by an entirely different mechanism, namely by stabilizing expression of TMEM16A in the plasma membrane. In contrast to small molecule activators, which were unable to activate endogenous TMEM16A currents, exposure of the cells to a CLCA1-enriched media strongly augmented endogenous TMEM16A currents (Figure 7A,B). Thus, the search for activators or potentiators of TMEM16A should also consider the activatory pathway described for CLCA1.

## 3. Discussion

**Figure 8:**
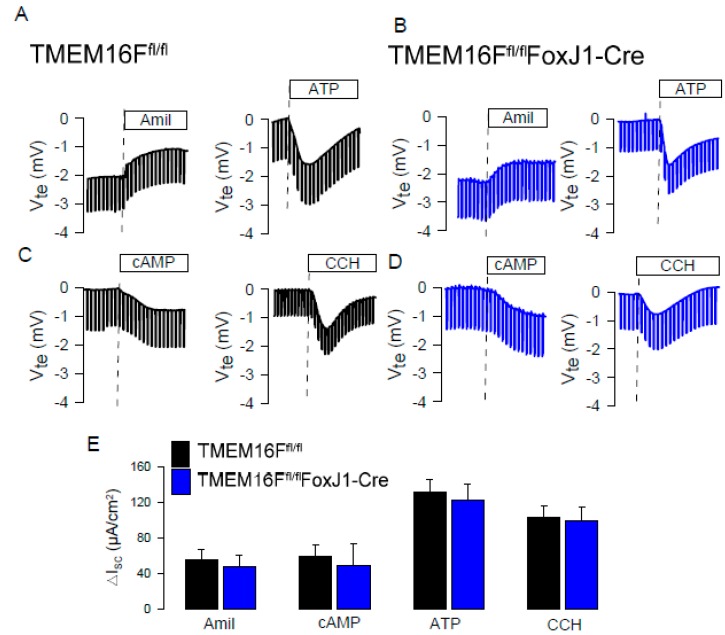
TMEM16F does not contribute to ion transport in mouse airways. Original Ussing chamber recordings obtained under open circuit conditions in tracheas from TMEM16F^fl/fl^ (wild type) (**A**,**C**) and TMEM16F^fl/fl^FoxJ1Cre (TMEM16F knockout in ciliated airway epithelial cells) (**B**,**D**) mice. Inhibition of epithelial Na^+^ absorption (via ENaC) by amiloride (10 µM), activation of CFTR by IBMX and forskolin (100 µM and 2 µM; cAMP), and activation of Ca^2+^-dependent Cl^−^ secretion by luminal ATP (100 µM) and basolateral carbachol (CCH; 100 µM) were comparable in wild type and TMEM16F-knockout tracheas. (**E**) Summaries of the calculated short circuit currents. Mean ± SEM (4 tracheas for each series).

The present study along with our previous data clearly indicate that TMEM16A is the relevant CaCC in intestinal and airway epithelial cells (Figure 1) [26,38,39,40]. When analyzing TMEM16F knockout mice, we also found no evidence for a contribution of TMEM16F to CaCC in airway or intestinal epithelium [26] (Figure 8). We compared the effects of various drugs on endogenous and overexpressed TMEM16A. Patch clamp experiments with a cytosolic-like buffer solution at 37 °C [41], and YFP-quenching experiments unmasked spontaneous activity of overexpressed human TMEM16A/F at basal (0.1 µM) intracellular Ca^2+^ (Figure 2) [21]. No spontaneous currents were observed in mock transfected HEK293 cells [21], or HT_29_ cells. In an earlier study we analyzed potential mechanisms for the spontaneous activity of TMEM16A, and found that activation is suppressed by inhibition of phospholipase A2 (PLA_2_) [21]. Earlier results also demonstrated activation of overexpressed TMEM16A/F by the bee toxin melittin, which stimulates PLA_2,_ and by direct application of lysophospholipids (LPL) [21,42]. In the present study, neither melittin nor LPL or other phospholipids activated endogenous TMEM16A (Figure 5). In the absence of intracellular Ca^2+^, TMEM16A/F currents were completely absent, indicating that binding of Ca^2+^ is a prerequisite for basal activity and for the activation by different drugs [21]. We speculate that intracellular Ca^2+^ ions may have a better access to overexpressed than to endogenous TMEM16A/F. Using YFP-quenching, we examined the effects of the activators melittin (1 µM) and cinnamaldehyde (10 µM) on endogenous TMEM16A in HT_29_ cells in the presence of Ca^2+^ increasing CPA (1 µM). However, we did not find activation of CaCC. These results again suggest that overexpressed TMEM16A responds differently to potential activators. Overexpressed and endogenous TMEM16A may be localized in different plasma membrane compartments or, alternatively, accessory proteins are missing in overexpressing cells, that normally keep the channel closed under resting [Ca^2+^]_i_.

Along this line, overexpressed TMEM16A was shown to be directly activated by the inositolphosphate INO-4995, while INO-4995 potentiated rather than activated endogenous TMEM16A [20,43]. Similar was observed in the present study with diC8-PIP_2_, which activated TMEM16A overexpressed in HEK293 cells, but had little effects on endogenous TMEM16A in HT_29_ cells (Figure 4 and Figure 5). Our results correspond to previous publications on the regulation of TMEM16A by PIP_2_: TMEM16A expressed endogenously in rat arterial smooth muscle cells was not activated but rather inhibited by diC8-PIP_2_ [44], while TMEM16A overexpressed in HEK293 cells was activated [18,19].

Similar differential activation of endogenous vs. exogenous TMEM16A was observed for the compound Eact. Eact has been reported as an activator of TMEM16A [15], but was suggested recently to activate TMEM16A indirectly by inducing Ca^2+^ influx through activation of TRPV1/4 [16,45]. Currently, Eact is one of the few accessible TMEM16A activators and therefore deserved further analysis: (i) Although TRPV4 is expressed in HT_29_ cells, Eact and the TRPV4-activator GSK1016790 did not activate TMEM16A. Eact caused only a very small increase in intracellular Ca^2+^ (Figure 1). (ii) TMEM16A overexpressed in HEK293 cells was fully activated by Eact, while Eact-induced Ca^2+^ increase was only very small (Figure 1 and Figure 5). (iii) Eact activated overexpressed TMEM16A and TMEM16F. The present data speak in favor of Eact directly activating overexpressed TMEM16A/F at basal [Ca^2+^]i. For potentiation of endogenous channels, pre-stimulation by a Ca^2+^-increasing agonist may be required, which is reminiscent to the potentiator ETX001 [17]. Cinnamaldehyde also activated overexpressed TMEM16A, with little effects on endogenous channels. The current results may be relevant when choosing the appropriate cellular system for drug testing or high throughput screening. 

The present study also compared inhibition of endogenous and overexpressed TMEM16A. Here the results were more consistent, i.e., the inhibitors examined here (benzbromarone, niclosamide, Ani9) blocked both endogenous and overexpressed channels (Figure 2). There are, however, issues regarding the specificity of these inhibitors. We reported earlier that niclosamide, apart from inhibiting TMEM16A, also inhibits TMEM16F [25]. Similar is shown in the present report for benzbromarone. Ani9 [30] showed some effects on time-dependent activation of TMEM16F currents (Figure 2). Ani9 did not block ATP-induced Ca^2+^ rise in HT_29_ cells, but showed some inhibitory effects on [Ca^2+^]_i_ in Cal33 and M1 cells (Figure 2). In contrast, benzbromarone and niclosamide clearly inhibited Ca^2+^ increase by stimulation with ATP (Figure 2). The data provide evidence that niclosamide lowers the Ca^2+^ store content and dampens Ca^2+^ influx (Figure 3). Nevertheless, when intracellular Ca^2+^ was clamped to 1 µM, niclosamide consistently inhibited TMEM16A (Figure 3A,B). The fact that TMEM16A enhances local Ca^2+^ signals by tethering the IP_3_ receptor complicates the analysis [22,23,26,46]. Moreover, some TMEM16A compounds were shown by Bill et al. to result in TMEM16A degradation, which may contribute to inhibition of Ca^2+^ signals [47]. Alternatively, it is conceivable that TMEM16A inhibitors that modulate ATP-induced intracellular calcium, could also interfere with other TMEM16A interacting partners to modulate channel activation and increase [Ca^2+^]i. Thus, Liu et al. (2020) found endophilin A2 regulated TMEM16A by autophagy-mediated degradation [48].

This somewhat convoluted situation calls for attention when interpreting physiological effects of inhibitors and activators of TMEM16A. In addition, voltage clamp experiments with voltage-gated TMEM16A channels may provide different results than experiments under non-voltage clamped conditions (YFP quenching). It is therefore indispensable to confirm results with novel small molecules by using knockdown technologies *in vitro* and TMEM16A-knockout animals [5,25,49]. The diversity of molecules that have been reported to inhibit or activate TMEM16A raises questions as to their specificity. As hypothesized earlier, interference of these molecules with plasma membrane lipids may be part of the mechanism of action. The present data as well as recent reports on the activation of the channel by lipid peroxidation [50], PLA_2_ [21,42], inositolphosphates such as INO-4995 [20], PIP_2_, cholesterol, and fatty acids [18,51] strongly support a regulation of TMEM16A by plasma membrane lipids [21].

## 4. Material and Methods

### 4.1. Knockout Animals

Generation of mice with a floxed TMEM16F allele was described previously [52]. For KO of TMEM16F in ciliated airway epithelial, TMEM16Ffl/fl mice were crossed with FoxJ1-Cre mice [53]. All animal experiments were approved by the local ethics committee of the Government of Unterfranken/Würzburg (AZ: 55.2-2532-2-328, approved on 3 April 2017) and were conducted according to the guidelines of the American Physiological Society and the German law for the welfare of animals.

### 4.2. Cell Culture

All cells were grown at 37 °C in a humidified atmosphere with 5% (*v*/*v*) CO_2_. HT_29_ human colonic carcinoma epithelial cells stably overexpressing iodide-sensitive enhanced yellow fluorescent protein (eYFP-I152L; HT_29_-YFP) were cultured in McCoy’s 5A medium supplemented with 10% (*v*/*v*) fetal bovine serum (FBS) and 1 mg/mL G418 selection antibiotic (all from Capricorn Scientific, Ebsdorfergrund, Germany). Human embryonic kidney HEK293 stably coexpressing TMEM16A and eYFP-I152L (HEK-TMEM16A-YFP) were grown in DMEM low glucose medium supplemented with 10% (*v/v*) FBS, 1% (*v/v*) L-glutamine 200 mM and 10 mM HEPES (all from Capricorn Scientific, Ebsdorfergrund, Germany), in the presence of selection antibiotics puromycin 0.5 µg/mL (Sigma-Aldrich Chemie GmbH, 82024 Taufkirchen, Germany) and hygromycin B 150 µg/mL (Capricorn Scientific, Ebsdorfergrund, Germany). Mouse collecting duct cell line (M1) was cultured in DMEM/F12 medium supplemented with 5% (*v/v*) fetal bovine serum (FBS), 1% (*v/v*) insulin-transferrin-selenium (ITS) 100×, and 1% (*v/v*) l-glutamine 200mM (all from Capricorn Scientific, Ebsdorfergrund, Germany). Squamous cell tongue carcinoma cell line Cal-33 was cultured in DMEM low glucose medium supplemented with 10% (*v/v*) FBS (all from Capricorn Scientific, Ebsdorfergrund, Germany).

Construction of expression plasmids has been described previously [21]. HEK293 cells were seeded in fibronectin- and collagen-coated 18-mm coverslips and transfected with bicistronic IRES plasmid vectors encoding hTMEM16A, hTMEM16F, or empty pcDNA3.1 vector (mock) and CD8 for detection of transfected cells by binding of anti-CD8 labeled beads, using standard protocols for Lipofectamine 3000. All experiments were performed 48–72 h after transfection.

Knockdown of TMEM16 proteins in HT29-YFP cells was performed through transfection by electroporation of siTMEM16A or siTMEM16F (both from Invitrogen, Carlsbad, CA, USA), using a neon transfection system (Invitrogen, Carlsbad, CA, USA) with a program of 3 pulses, 1650 V, 10 ms. Alternatively, the siRNAs were transfected using standard protocols for Lipofectamine 3000 (Invitrogen, Carlsbad, CA, USA). Scrambled siRNA (Invitrogen, Carlsbad, CA, USA) was transfected as control. All cells transfected with siRNA were used for experiments 72 h after transfection.

### 4.3. RT-PCR

For RT-PCR total RNA from cell lines was isolated using NucleoSpin RNA II columns (Macherey-Nagel, Düren, Germany). Total RNA (1 µg/50 µL reaction) was reverse-transcribed using random primers and M-MLV Reverse Transcriptase RNase H Minus (all from Promega, Mannheim, Germany). Each RT-PCR reaction contained sense and antisense primers (0.5 µM) (Table 2), 0.5 µL cDNA, and GoTaq Polymerase (all from Promega, Mannheim, Germany). After 2 min at 95 °C, cDNA was amplified (25–35 cycles for target sequence and 25 cycles for the reference GAPDH) for 30 s at 95 °C, 30 s at 56 °C, and 1 min at 72 °C. PCR products were then visualized by loading on peqGREEN- (Peqlab, Düsseldorf, Germany) -containing agarose gels and analyzed using ImageJ.

### 4.4. Western Blotting

Protein was isolated from cells using a sample buffer containing 50 mM Tris-HCl, 150 mM NaCl, 50 mM Tris, 100 mM dithiothreitol, 1% Nonidet P-40, 0.5% sodium deoxycholate, and 1% protease inhibitor mixture (Sigma-Aldrich, St. Louis, MO, USA). Proteins were separated by 7.5% SDS-PAGE and transferred to a PVDF membrane (GE Healthcare, Munich, Germany). Membranes were incubated with primary rabbit polyclonal anti-TMEM16A DOG1 antibody (#NBP1-49799, Novus Biologicals, Centennial, CO, USA; 1:500 in 1% (*w/v*) NFM/TBS-T) overnight at 4 °C. The CFTR antibody was from Alomone Labs (Israel). Membranes were then incubated with horseradish peroxidase (HRP)-conjugated goat anti-rabbit secondary antibody at RT for 2 h and immunoreactive signals were visualized using supersignal chemiluminescence substrate detection kit (Pierce Biotechnology, Rockford, IL, USA). Actin (1:10,000) was used as a loading control.

### 4.5. Patch Clamping

Cells were grown on fibronectin and collagen-coated glass coverslips for patch clamp experiments. Coverslips were mounted in a perfused bath chamber on the stage of an inverted microscope (IM35, Zeiss, Oberkochen, Germany) and kept at 37 °C. Patch pipettes were filled with a cytosolic-like solution containing in mM: KCl 30, K^+^-Gluconate 95, NaH_2_PO_4_ 1.2, Na_2_HPO_4_ 4.8, EGTA 1, Ca^2+^-Gluconate 0.758, MgCl_2_ 1.03, D-Glucose 5, ATP 3, pH 7.2. The Ca^2+^ activity was 0.01, 0.1, or 1 μM. The bath was perfused continuously with Ringer solution at a rate of 8 mL/min. Patch clamp experiments were performed in the fast whole cell configuration. Patch pipettes had an input resistance of 2–4 MΩ when filled with cytosolic-like solution. The access conductance was monitored continuously and was 60–140 nS. Currents (voltage clamp) and voltages (current clamp) were recorded using a patch clamp amplifier (EPC 7, List Medical Electronics, Darmstadt, Germany), the LIH1600 interface and PULSE software (HEKA, Lambrecht, Germany) as well as Chart software (AD Instruments, Spechbach, Germany). Data were stored continuously on a computer hard disc and analyzed using PULSE software. In regular intervals, membrane voltage (Vc) was clamped in steps of 20 mV from −100 to +100 mV from a holding voltage of −100 mV. Current density was calculated by dividing whole cell currents by cell capacitance.

### 4.6. Iodide-Sensitive YFP-Quenching Assay of Anion Conductance/TMEM16A Activity

Cells stably transfected with iodide-sensitive YFP were plated in transparent, 96-well plates (Sarstedt, Nümbrecht, Germany), cultured 24–72 h to 80–90% confluence, washed with gluconate substituted-Ringer solution (mmol/L: NaCl 100–120; Na^+^-gluconate 20–40; KCl 5; MgCl_2_ 1; CaCl_2_ 2; D-Glucose 10; HEPES 10), and incubated with or without test compounds in this solution. Total intracellular YFP fluorescence intensity in each well was measured in a fluorescence microplate reader (NOVOstar, BMG Labtech, Ortenberg, Germany) kept at 37 °C, using an excitation wavelength of 485 nm and emission detection at 520 nm. Fluorescence was read continuously during injection of an iodide (I^−^)-substituted Ringer solution (mmol/L: NaCl 100–120; NaI 20–40; KCl 5; MgCl_2_ 1; CaCl_2_ 2; D-Glucose 10; HEPES 10) by a syringe pump and following injections of a symmetrical Ringer solution (in mmol/L: NaCl 100–120; Na^+^-Gluconate 10–20; NaI 10–20; KCl 5; MgCl_2_ 1; CaCl_2_ 2; D-Glucose 10; HEPES 10) carrying test compounds. Original data were collected, background fluorescence was subtracted, and the initial rate of maximal fluorescence decay caused by I^-^ influx/YFP fluorescence-quenching upon acute injection (or pre-incubation) of test compounds was measured to determine anion conductance/activity of TMEM16A.

### 4.7. Ca^2+^ Measurements

Cells were seeded on glass cover slips and loaded with 2 µM Fura-2/AM and 0.02% pluronic F-127 (Invitrogen, Darmstadt, Germany) in Ringer solution (mmol/L: NaCl 145; KH_2_PO_4_ 0,4; K_2_HPO_4_ 1,6; Glucose 5; MgCl_2_ 1; Ca^2+^-Gluconate 1,3) for 1 h at room temperature. Fluorescence was detected in cells perfused with Ringer’s solution at 37 °C using an inverted microscope (Axiovert S100, Zeiss, Oberkochen, Germany) and a high speed polychromator system (VisiChrome, Puchheim, Germany). Fura-2 was excited at 340/380 nm, and emission was recorded between 470 and 550 nm using a CoolSnap camera (CoolSnap HQ, Visitron). [Ca^2+^]*_i_* was calculated from the 340/380 nm fluorescence ratio after background subtraction. The formula used to calculate intracellular calcium ([Ca^2+^]*_i_)* was [Ca^2+^]*_i_ =Kd* × (*R* − *R*_min_)/(*R*_max_ − *R*) × (S_f2_/S_b2_), where *R* is the observed fluorescence ratio. The values *R*_max_ and *R*_min_ (maximum and minimum ratios) and the constant S_f2_/S_b2_ (fluorescence of free and Ca^2+^-bound Fura-2 at 380 nm) were calculated using 1 µmol/L ionomycin (Calbiochem, La Jolla, CA, USA), 5 µmol/L nigericin (Sigma-Aldrich, St. Louis, MO, USA), 10 µmol/L monensin (Sigma-Aldrich, St. Louis, MO, USA), and 5 mmol/L EGTA to equilibrate intracellular and extracellular Ca^2+^ in intact Fura-2-loaded cells. The dissociation constant for the Fura-2•Ca^2+^ complex was taken as 224 nmol/L. Control of experiment, imaging acquisition, and data analysis were done with the software package Meta-Fluor (Universal Imaging, New York, NY, USA).

### 4.8. Data and Statistical Analysis

If not indicated otherwise, all chemicals were from Sigma-Aldrich. Data are shown as individual traces or as summaries with mean values ± SEM and number of experiments or cells given in each figure’s legend. For statistical analysis, paired or unpaired Student’s *t*-test was used as appropriate. A *p*-value < 0.05 was accepted as statistically significant difference (indicated by # for unpaired data and by * for paired data).

## Figures and Tables

**Table 1:** Effects of potential activators/potentiators of TMEM16A in HT29 cells (endogenous TMEM16A) and HEK293 cells (overexpressed TMEM16A).

Compound	Endogenous		Expressed	
	Activation	Potentiation	Activation	Potentiation
Eact	No (50 µM; *n* = 5)	No (50 µM; *n* = 5)	Yes (10 µM; *n* = 5)	Yes (10 µM; *n* = 5)
GSK1016790A	No (50 µM; *n* = 5)	No (50 µM; *n* = 5)		
Melittin	Yes (2 µM; *n* = 5)	Yes (2 µM; *n* = 5)	Yes (0.5 µM; *n* = 5)	Yes (0.5 µM; *n* = 5)
Cinnamaldehyde	No (1 µM; *n* = 5)	No (1 µM; *n* = 5)	Yes (1 µM; *n* = 5)	Yes (0.5 µM; *n* = 5)
diC8-PIP2-PL	No (50 µM; *n* = 5)	No (50 µM; *n* = 5)	Yes (50 µM; *n* = 5)	Yes (50 µM; *n* = 5)
CLCA1	No	Yes		

**Table 2:** RT-PCR primers.

Target	Primers	PCR Product
TMEM16A	forward: 5’-CGACTACGTGTACATTTTCCG	445 bp
	reverse: 5´-GATTCCGATGTCTTTGGCTC	
TMEM16F	forward: 5´-GGAGTTTTGGAAGCGACGC	325 bp
	reverse: 5´-GTATTTCTGGATTGGGTCTG	
TRPV4	forward: 5´-GCCCCACATTGTCAACTACC	490 bp
	reverse: 5´-CCGCAGCAGTTCATTGATGG	
GAPDH	forward: 5´-GTATTGGGCGCCTGGTCAC	200 bp
	reverse: 5´-CTCCTGGAAGATGGTGATGG	
